# Non-Stoichiometric Ba_x_Mn_0.7_Cu_0.3_O_3_ Perovskites as Catalysts for CO Oxidation: Optimizing the Ba Content

**DOI:** 10.3390/nano15020103

**Published:** 2025-01-10

**Authors:** Álvaro Díaz-Verde, Emerson Luiz dos Santos Veiga, Héctor Beltrán-Mir, María José Illán-Gómez, Eloísa Cordoncillo-Cordoncillo

**Affiliations:** 1 Carbon Materials and Environment Research Group, Inorganic Chemistry Department, Institute of Materials Science (IUMA), University of Alicante, Ctra San Vicente del Raspeig s/n, San Vicente dek Raspeig, 03690 Alicante, Spain; alvaro.diaz@ua.es; 2 Department of Inorganic and Organic Chemistry, University Jaume I, Av. Vicent Sos Baynat s/n, 12071 Castellón de la Plana, Spain; edossant@uji.es (E.L.d.S.V.); mir@uji.es (H.B.-M.); cordonci@qio.uji.es (E.C.-C.)

**Keywords:** perovskite, manganese, copper, CO oxidation, Ba non-stoichiometry

## Abstract

In this work, a series of Ba_x_Mn_0.7_Cu_0.3_O_3_ samples (x: 1, 0.9, 0.8, and 0.7, BxMC) was synthesized, characterized, and used as catalysts for CO oxidation reaction. All formulations were active for CO oxidation in the tested conditions. A correlation between the electrical conductivity, obtained by impedance spectroscopy, and the reducibility of the samples, obtained by H_2_-TPR, was observed. The Ba_0.8_Mn_0.7_Cu_0.3_O_3_ composition (B0.8MC) showed the best catalytic performance (comparable to that of the 1% Pt/Al_2_O_3_ reference sample) during tests conducted under conditions similar to those found in the exhaust gases of current gasoline engines. The characterization data suggest the simultaneous presence of a high Mn(IV)/Mn(III) surface ratio, oxygen vacancies, and reduced copper species, these two latter being key properties for ensuring a high CO conversion percentage as both are active sites for CO oxidation. The reaction temperature and the reactant atmosphere composition seem to be the most important factors for achieving a good catalytic performance, as they strongly determine the location and stability of the reduced copper species.

## 1. Introduction

The ongoing efforts to reduce the emissions of pollutant gases generated by the industrial and automotive sectors motivate the development of significant advancements in catalyst technologies. Carbon monoxide (CO), a colorless, odorless, and highly toxic gas, is a major pollutant generated by the incomplete combustion of carbon-containing fuels, such as those traditionally used in automobile engines and industrial processes [1,2]. As is well known, its toxicity arises from its ability to bond irreversibly with hemoglobin, reducing the capacity of the body to transport oxygen, which leads to severe health effects (dizziness, confusion, and even death at very high concentrations [3,4]). Consequently, the scientific community is developing deep research into the conversion of CO through a catalytic process. Regarding CO oxidation catalysts, noble metal-based catalysts (like Pt) are highly active [5,6,7], but they present, as two main handicaps, a high cost and susceptibility to sintering at elevated temperatures [8,9]. Thus, currently, the focus has shifted towards developing cost-effective and stable alternative catalysts. Transition metal oxides, such as CuO, Co_3_O_4_, CeO_2_, and MnO_2_, have emerged as promising candidates due to their lower cost and acceptably high catalytic activity [10,11,12,13]. Thus, in recent studies carried out under real automobile exhaust conditions, such as cold start scenarios, new catalyst designs, such as MnCo_2_O_4_ supported on dealuminated zeolite molecular sieves [14], have been proposed. Furthermore, these new formulations can also be tuned by the addition of other components, with the Cu-based ones the most used for the CO removal application. This is because the incorporation of CuO improves the redox and the catalytic properties of the raw solids as a consequence of both the interaction between the Cu(II)/Cu(I) redox pair with other reducible metals and of its ability to activate the CO molecule [15,16,17,18].

Perovskite-type oxides (ABO_3_) are solids characterized by their affordability, high stability, and excellent optical, magnetic, electronic, and ferroelectric properties [19]. These mixed oxides also exhibit intriguing catalytic properties, mainly determined by the metal in the B position, that can be tunable by adjusting their composition [20,21,22,23,24,25,26,27,28]. Thus, several studies have proved that the catalytic activity of perovskites can be significantly enhanced by incorporating transition metals as dopants into the B position, as they improve their redox properties through changes in the oxidation state of the raw B metal, and due to the generation of oxygen vacancies, which could be active sites for the activation of different reactants [20,21]. In addition, it has been proposed in the literature that the modification of the raw species located at the A site (by doping or changing their stoichiometry) seems to also indirectly improve the catalytic properties of perovskites, although they usually are non-reducible components. Thus, the main factors that can be modified by changing the perovskite formulations in the A site are the amount of oxygen vacancies, the ionic mobility, and the oxidation state of the metals in the B position [22,23,24,25,26,29].

Considering the above-described ability of Cu to enhance the redox properties of Ba/Mn perovskites [30], the doping of the perovskite-type samples with Cu has been previously contemplated in order to improve the catalytic performance of these solids. Thus, Torregrosa-Rivero et al. [31] developed a series of BaMn_1−x_Cu_x_O_3_ perovskites that were tested as catalysts for the NO_2_-assisted soot oxidation reaction, concluding that the BaMn_0.7_Cu_0.3_O_3_ sample featured the highest conversion at low temperatures while also presenting one of the highest CO_2_ selectivities. Based on this conclusion, but also on the improvement in the catalytic performance for CO oxidation of a series of Ba_x_MnO_3_ (x < 1) perovskites (due to the increase in the amount of oxygen vacancies, the reducibility, and the improved oxygen mobility) [32], in this paper, a series of Ba_x_Mn_0.7_Cu_0.3_O_3_ (x = 1, 0.9, 0.8, and 0.7) perovskite formulations has been synthesized, characterized, and used as catalysts for CO oxidation in conditions simulating those currently found in the Three-Way Catalysts (TWCs) exhaust. The selection of the Ba_x_Mn_0.7_Cu_0.3_O_3_ formulations in this paper is done with the aim to obtain new insights into the relationship between the composition of the catalysts and their catalytic activity, since in the literature most of the papers focused on the presented application predominantly analyze the substitution of the A metal by other elements, instead of changing the A metal percentage. Thus, in this study, the advantages of doping the B position of the perovskite structure with Cu and of decreasing the Ba content will be analyzed with the aim of identifying a synergy that leads to an improved catalytic performance.

## 2. Materials and Methods

### 2.1. Synthesis and Characterization of Ba_x_Mn_0.7_Cu_0.3_O_3_ Samples

The synthesis of Ba_x_Mn_0.7_Cu_0.3_O_3_ (BxMC) samples was performed via the sol–gel method adapted to an aqueous medium [33,34]. Firstly, citric acid (C_6_H_8_O_7_, Sigma-Aldrich, St. Louis, MO, USA, 99.0% wt, used as gelling and chelating agent in a 1:2 Mn + Cu:citric acid molar ratio) was dissolved at 60 °C in 40 mL of distilled water. Barium acetate (Ba(CH_3_COO)_2_, Sigma-Aldrich, 99.0% wt), manganese(II) nitrate tetrahydrate (Mn(NO_3_)_2_ · 4 H_2_O, Sigma-Aldrich, 99.0% wt) and copper(II) nitrate trihydrate (Cu(NO_3_)_2_ · 3 H_2_O, Panreac, 99.0% wt) were added as metal precursors. Later, the gel formation was carried out at 65 °C. The pH was maintained at 9 during the process by employing an ammonia solution (NH_3_, Panreac, Castellar del Valles, Spain, 30% wt). Finally, the gel was dried at 90 °C for 48 h, and the solids were calcined at 850 °C for 6 h.

The copper content analysis was conducted by the ICP-OES technique, using a Perkin–Elmer device model Optimal 4300 DV. The sample preparation involved a mineralization using a diluted aqua regia solution (HNO_3_:HCl, 1:3) and stirring at 60 °C for 1 h.

The surface area of the samples was analyzed by N_2_ adsorption at −196 °C employing an Autosorb-6B instrument from Quantachrome (Anton Paar Austria GmbH, Graz, Austria). The degassing of the samples was carried out at 250 °C for 4 h prior to the experiments.

The crystalline structure of the samples was determined using X-ray diffraction (XRD). The X-ray patterns were obtained between 20° and 80° 2θ angles with a step rate of 0.4° min^−1^ using Cu K_α_ (0.15418 nm) radiation in a Bruker D8-Advance device. Average crystal sizes [35] and lattice strain [36] values of the perovskite phases were obtained by the Williamson–Hall method, calculated by the determination of the Y interception and the slope of regression line, respectively, as described by Equation (1).

(1) $$B\cos\theta =\varepsilon \left(4\sin\theta \right)+\frac{K\lambda }{D}$$

where B is the full width at half maximum of the XRD peaks, ε is the lattice strain, K is the shape factor (equal to 0.9), λ is the Cu K_α_ wavelength, and D is the average crystal size. Finally, XRD refinement was conducted to quantify the percentages of the detected crystalline phases using the HighScore Plus software (Malvern Panalytical B.V., Almelo, The Netherlands, 4.9 (4.9.0.27512) version).

The surface of the samples was studied by X-ray Photoelectron Spectroscopy (XPS) employing a K-Alpha Photoelectron Spectrometer by Thermo-Scientific (Waltham, MA, USA) with an Al K_α_ (1486.7 eV) radiation source. To obtain XPS spectra, the pressure of the analysis chamber was maintained at 5 × 10^−10^ mbar. The binding energy (BE) and kinetic energy (KE) scales were calibrated by setting the C 1s transition at 284.6 eV, and the deconvolution of the resulting XPS profiles was performed with the Thermo Avantage software (v5.9929). During the experiments, the following transitions were analyzed: O 1s, Mn 2p^3/2^, Mn 3p, Cu 2p^3/2^, Cu L_3_M_4.5_M_4.5_ Auger signal, and Ba 3d^5/2^.

The reducibility of the catalysts was analyzed by Temperature-Programmed Reduction with H_2_ (H_2_-TPR) in a Pulse Chemisorb 2705 (from Micromeritics, Norcross, GA, USA) equipped with a Thermal Conductivity Detector (TCD). During the experiments, 30 mg of sample were heated at 10 °C min^−1^ from 25 °C to 1000 °C in 5% H_2_/Ar atmosphere (40 mL min^−1^). The amount of consumed H_2_ was determined using a copper (II) oxide (CuO, Sigma-Aldrich, 99.9% wt) reference sample, which is known to be reduced according to reaction (2) [37].

CuO + H_2_ → Cu + H_2_O (2)

O_2_-TPD experiments were conducted with a Thermo Gravimetric Mass Spectrometer (TG-MS) system (Q-600-TA and Thermostar from Balzers Instruments, Balzers Liechtenstein (Pfeiffer Vacuum GmbH, Asslar, Germany), respectively). During the analyses, 16 mg of sample were heated at 10 °C min^−1^ from room temperature to 950 °C under a 100 mL min^−1^ of He. Prior to the experiments, each sample was subjected to a 1 h preheating treatment at 150 °C to remove the moisture. During the experiments, the 32 *m*/*z* signal was monitored for the O_2_ evolved. The quantification of the evolved oxygen was performed using a CuO reference sample, which decomposes to Cu_2_O under the tested conditions [38], according to reaction (3).

4 CuO → 2 Cu_2_O + O_2_ (3)

Impedance spectroscopy (IS) measurements were conducted to determine the electrical response of the samples. IS is a powerful technique that allows the investigation of the kinetics of electrochemical reactions and of the electrical properties of materials and their interfaces. Considering that catalysis phenomena are largely dependent on the surface, IS proves especially valuable for studying interactions at the solid–gas interface. Thus, IS can be used to investigate catalytic processes by evaluating charge carrier mobility within bulk or interface regions and understanding the role of defects in the material conductivity [39,40,41]. To perform IS measurements, the sample must be analyzed using a frequency range from 10 Hz to 10 MHz at various temperatures to distinguish the regions contributing to the total sample resistance. By applying a voltage across the sample and recording the current response, the resistive (R) and capacitive (C) elements of the impedance can be determined. Each region within the sample has unique relaxation times and time constants, τ, defined as the product of R and C (Equation (4)). This allows for separation of sample regions on a frequency scale based on the relaxation time of each, as shown in Equation (5).

(4) τ=RC

(5) $$\omega_{max}RC=1$$

where ω_max_ is the frequency corresponding to the maximum loss, expressed by the formula ω_max_ = 2πf_max_ [42]. In ceramic materials, most electrically distinct regions can be represented by separate parallel RC components. Each parallel RC component corresponds to a semicircle on complex plane plots of Z*, which can also be represented in three other complex formalisms: admittance (Y*), electric modulus (M*), and permittivity (ε*). These formalisms are related through the following relationships: Z* = 1/Y*; M* = jωC_0_Z*; ε* = 1/M*; Y* = jωC_0_ε*, where C_0_ denotes the vacuum capacitance of the conductive cell. Each formalism includes real and imaginary parts, typically expressed as Z* = Z′ − jZ″; M* = M′ + jM″; ε* = ε′ − jε″; and Y* = Y′ + jY″ [43]. Using these equations, the data can be displayed as complex plane plots (often referred to as Nyquist plots), such as Z″ versus Z′, or as spectroscopic plots showing the imaginary components Z″ and M″ against log f. In the representation of Z″ and M″ versus log f, a Debye peak arises, which can be described by Equations (6) and (7):

(6) $$Z\prime \prime =R\left[\frac{\omega RC}{1+{\left(\omega RC\right)}^{2}}\right]$$

(7) $$M\prime \prime =\frac{\varepsilon_{0}}{C}\left[\frac{\omega RC}{1+{\left(\omega RC\right)}^{2}}\right]$$

where ε_0_ is the permittivity of free space, 8.854 × 10^−14^ F cm^−1^. Since ω_max_ is associated to the maximum of the Debye peak for each RC element in parallel, from Equations (6) and (7) we can obtain M″_max_, ${M\prime \prime }_{\max}=\frac{\varepsilon_{0}}{2C}$ and Z″_max_, ${Z\prime \prime }_{max}=\frac{R}{2}$. These Z″/M″ spectroscopic plots clarify the presence of different components that are involved in the overall electrical response, especially in those cases where the identification of the different regions of a sample is not well defined in the impedance complex plane plots, Z*. In many cases, a semicircle is not clearly observed for each region of the sample, resulting in distorted semicircles that make it difficult to identify the different regions. In addition to spectroscopic representations of Z″ and M″, log C′ versus log f plots can offer direct insights into the specific region being analyzed within a given frequency range. Capacitance primarily depends on the thickness of each material region (C = ε A/d, where ε is the absolute permittivity [ε′⋯ε_0_] with ε_0_ being the permittivity of free space, 8.854 × 10^−14^ F cm^−1^, A the cross-sectional area of the region, and d the thickness), causing the magnitude of C′ (C′ ≡ ε′C_0_) to vary significantly between regions. For instance, the bulk of the material typically has capacitance values around 10^−12^ F cm^−1^, while the grain boundaries range from 10^−11^ to 10^−9^ F cm^−1^, and the sample–electrode interface lies between 10^−6^ and 10^−5^ F cm^−1^.

In the context of the present paper, the IS technique can provide valuable information about the conductivity behavior of the samples, enabling a correlation between the presence of defects in the sample and its catalytic performance. During the experiments, the samples were uniaxially pressed into 5 mm diameter, 1 mm thick pellets using 5 wt.% PVB (Sigma-Aldrich) as a binder and subsequently sintered at 800 °C for 2 h. Opposite faces of the pellets were coated with electrodes made from platinum paste (Pt Ink 6082, Metalor), which was dried and decomposed by gradually heating to 800 °C. Finally, samples attached to the electrodes were placed in a conductivity jig and measured using an Agilent 4294A analyzer over the frequency range of 40 Hz–13 MHz with an AC voltage of 0.1 V. IS measurements were performed for BxMC samples from room temperature to 800 °C, under dry nitrogen atmosphere. These measurements were conducted in an inert atmosphere of nitrogen to prevent oxidation of the material and to better understanding the electrical response. The impedance data were corrected for overall pellet geometry.

### 2.2. Activity Tests

To determine the catalytic activity for CO oxidation, Temperature-Programmed Reaction experiments (CO-TPRe) were developed using three gas mixtures composed of: (i) 1% CO and 1% O_2_ in He, as an approximation of the CO partial pressure in the actual TWC working conditions [44]; (ii) 1% CO and 10% O_2_ in He, to analyze the effect of using a higher oxygen partial pressure in relation to (i); and (iii) 0.1% CO and 1% O_2_ in He, for determining the catalytic performance in a very low CO partial pressure in relation to (i). For these experiments, the samples (50 mg of catalyst and 100 mg of SiC loaded into a U-shaped quartz reactor) were subjected to heating at 10 °C min^−1^ until 500 °C in a 100 mL min^−1^ flow (Gas Hourly Space Velocity (GHSV) of 4967 h^−1^) of the gas mixture. Before the CO-TPRe tests, the catalyst–SiC mixture was preheated for 1 h at 600 °C in a 5% O_2_/He gas mixture to clean the surface of samples. Moreover, a commercial 1% Pt/Al_2_O_3_ sample (Sigma-Aldrich) was used as a reference, which was not subjected to the preheating treatment to minimize Pt sintering [45]. For the most active catalyst, stability tests consisting of two reaction cycles at 250 °C and 300 °C (5 h), using the gas mixtures (i) and (ii), were performed. Before each cycle, the catalyst was subjected to the preheating treatment described above (1 h at 600 °C in a 5% O_2_/He gas mixture). For reaction product determination, an Agilent 8860 Gas Chromatograph, equipped with a TCD and two packed columns (Porapak-Q and MolSieve-13X (Agilent Technologies Spain, Madrid, Spain)), was used. The percentage of CO conversion ($\chi_{CO}$) was calculated by Equation (8):

(8) $$\chi_{CO}\left(\%\right)=\frac{\left(CO_{in}-CO_{out}\right)}{CO_{in}}\cdot 100$$

where CO_in_ and CO_out_ are the inlet and outlet molar flow rates, respectively.

## 3. Results and Discussion

### 3.1. Chemical and Structural Properties

The BET surface area and the copper content (nominal and real obtained using the chemical formula and the ICP-OES data, respectively) of the samples are reported in Table 1. As expected for solids with almost negligible porosity, all mixed oxides present very low surface areas [46,47] which does not seem dependent on the chemical composition. On the other hand, the real and nominal copper content are quite similar, revealing that the sol–gel method allowed for achieving the required amount of copper for each sample.

**Figure 1:**
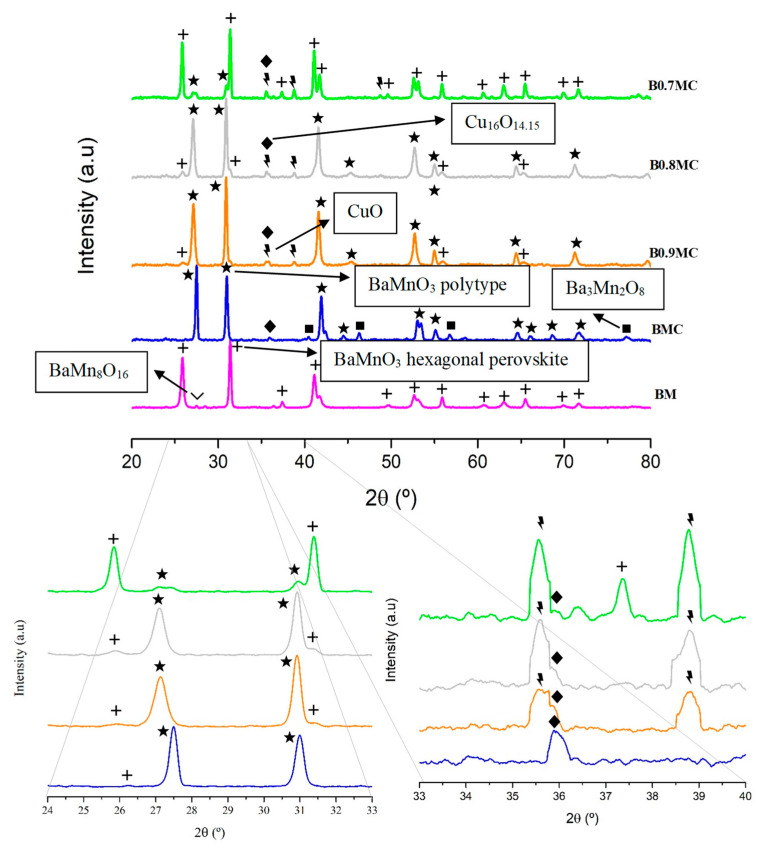
XRD patterns of BxMC samples and BM perovskite as reference.

X-ray diffraction profiles are shown in Figure 1, in which the one corresponding to a raw BaMnO_3_ perovskite (BM), previously studied by the authors [32] has been included for comparative purposes [48]. The two main crystalline phases detected are the BaMnO_3_ hexagonal perovskite structure (JCPDS-ICDD 26-0168) and the BaMnO_3_ polytype structure, which is a distortion of the hexagonal perovskite structure caused by the insertion of Cu into the perovskite lattice partially occupying the Mn ion position. This distortion is due to the deformation of the MnO_6_ octahedra due to the different size of Cu and Mn ions, which modifies the bond lengths [49]. Additionally, several diffraction peaks corresponding to three minority phases are identified: (i) the tenorite (CuO, JCPDS-ICDD 80-0076) phase, located at 2θ values of 35.58°, 38.80°, and 48.88°, whose intensity increases as the Ba content decreases; (ii) the copper oxide paramelaconite (Cu_16_O_14.15_, JCPDS-ICDD 71-0251), mainly for BMC sample and with a lower intensity for the Ba-deficient samples; and, finally, (iii) the Ba_3_Mn_2_O_8_ (JCPDS-ICDD 23-1026) phase, which only appears for BMC sample and that was previously detected for Ba_0.9_A_0.1_MnO_3_ (A = Mg and La) formulations [50]. Thus, the main crystalline phase for the BMC sample is the BaMnO_3_ polytype, but the intensity of the corresponding diffraction peaks decreases with decreasing Ba content (while simultaneously an increase in the intensity of the peaks corresponding to the hexagonal perovskite occurs [32]). Note that the same trend is followed by the cell parameters (see data in Table 2), suggesting that the transition from the polytype to the hexagonal structure takes place by the elongation of the length of the unit cell in the XY plane, which causes a decrease of the height. Furthermore, the size of the unit cell of the polytype structure slightly increases, the corresponding main diffraction peaks are shifted towards lower 2θ values from BMC to B0.8MC and, finally, the interplanar space values, calculated for the (110) plane, also increase. Note that, according to the data provided in Table 2 and due to the trend in the unit cell dimensions, the B0.7MC sample features the same parameters (cell parameters and (110) interplanar space) as those of the BM reference. This fact confirms that the change in the unit cell is due to the transition from a solid (BMC) where the polytype and the hexagonal phases coexist, to a solid (B0.7MC) for which the BaMnO_3_ hexagonal perovskite is the main crystalline phase. On the contrary, the average crystal sizes and the lattice strain values of the perovskite phase for BMC and BxMC samples (Table 2) do not follow a clear trend with the Ba content. Note that the coexistence of both crystalline phases in the presence of Cu causes the lattice strain values to be higher than those shown by the analogous series of perovskites in the absence of Cu (between 3·10^−5^ and 3·10^−4^ [32]). Finally, the structural change detected as the Ba content decreases is also found in the trend of the percentages corresponding to the crystalline phases, obtained by XRD refinement (Table 3). Regarding the Cu-based crystalline phases, the presence of a Cu_16_O_14.15_ phase (which also can be formulated as CuO_1−x_, with x ≈ 0.9) is observed, which is a metastable copper oxide with an intermediate composition between CuO and Cu_2_O [51,52,53]. Thus, the stability of this phase in the synthesized samples should be due to the strong interaction of the Cu species with the perovskite phase. In fact, copper oxide paramelaconite is found in BMC sample instead of the CuO phase; meanwhile CuO and Cu_16_O_14.15_ coexist in the Ba-deficient perovskites, with the amount of Cu_16_O_14.15_ lower as the Ba content decreases, as featured by the evolution of the shoulder at approximately 2θ = 36°.

The transition from the polytype structure to the hexagonal one could be caused by two different processes: (i) the decrease in Ba content makes the Cu insertion into the perovskite lattice more difficult, so the distortion of the structure becomes less pronounced and, in this case, the intensity of the diffraction peaks associated with CuO should increase; and/or (ii) the generation of lattice vacancies as the amount of Ba decreases allows Cu to occupy larger positions, resulting in a less pronounced distortion. In fact, the transition from Cu_16_O_14.15_ to CuO suggests that Cu species are located in sites with a lower interaction with the perovskite. However, the most accurate explanation will be suggested only after the analysis of the XPS results.

### 3.2. Surface Properties

**Figure 2:**
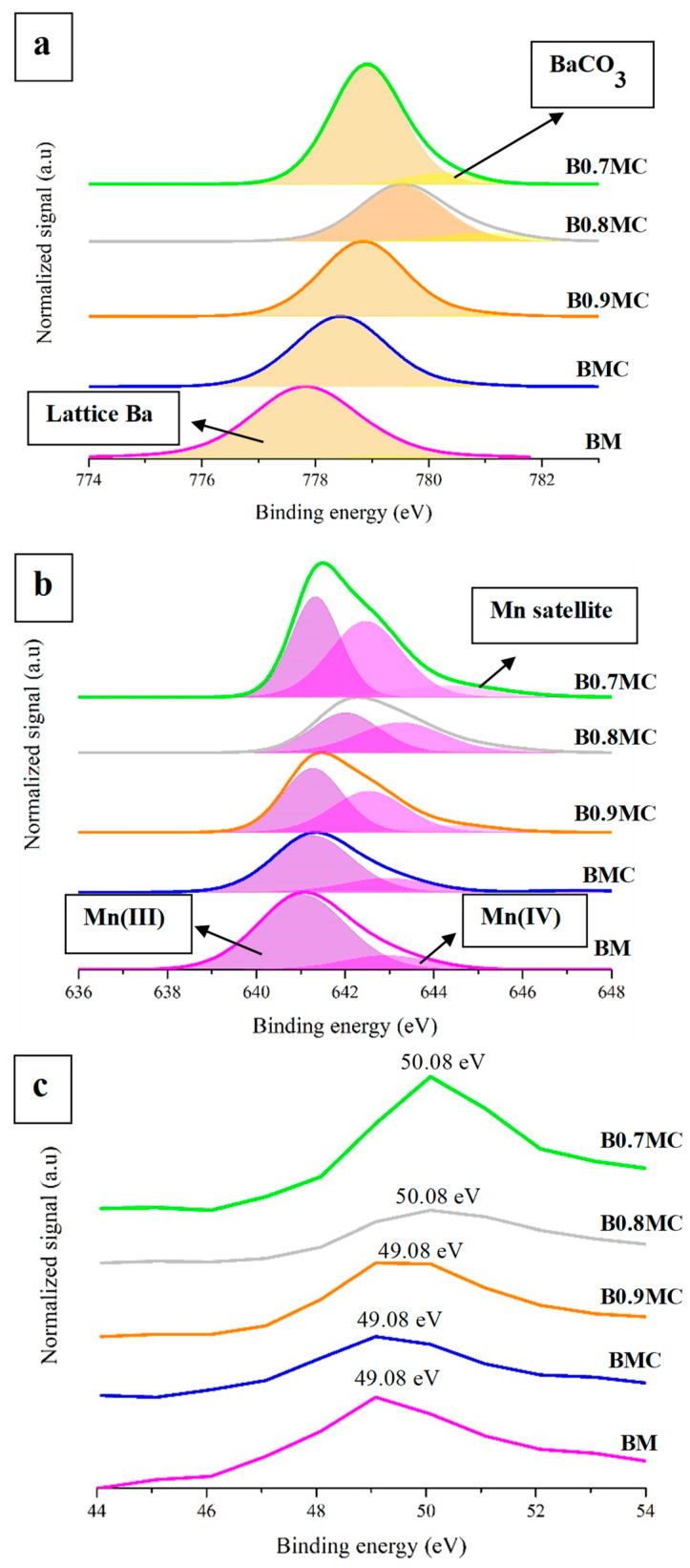

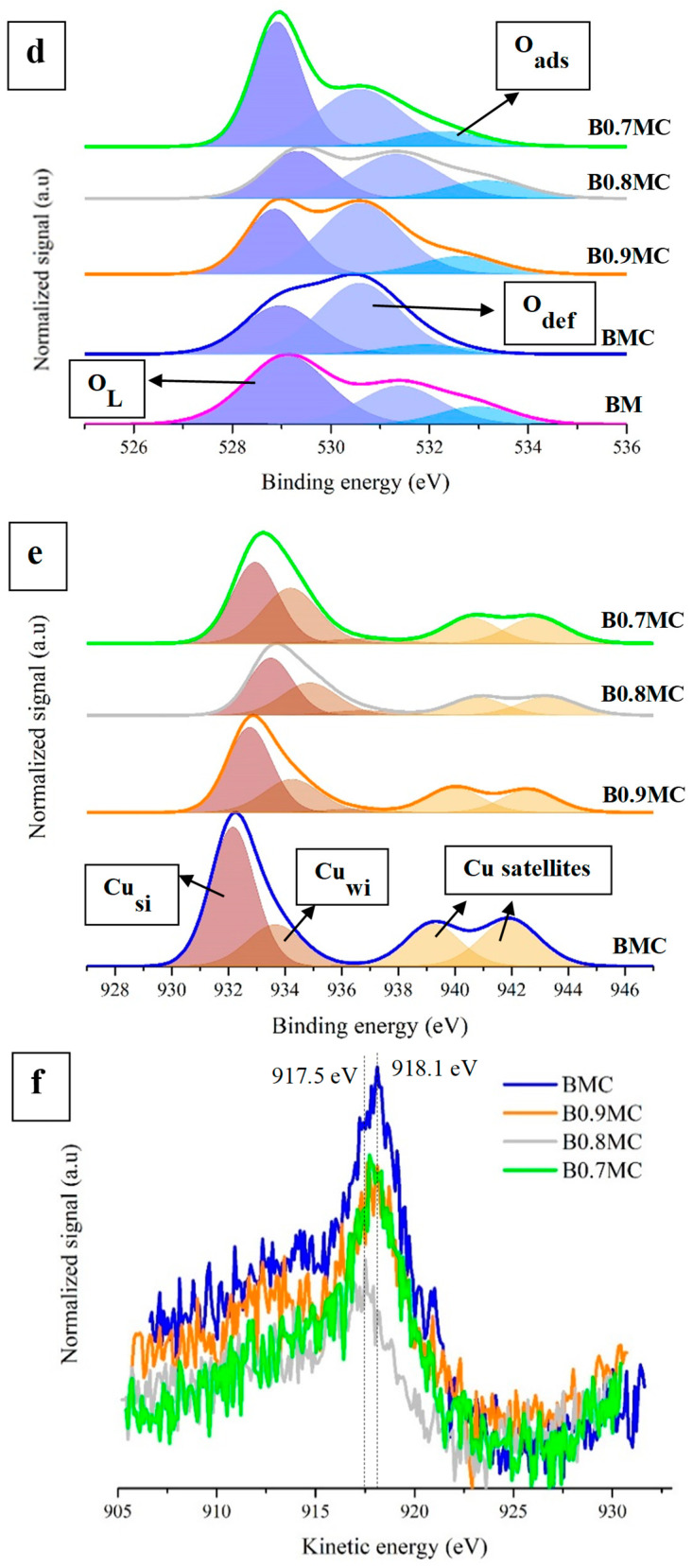
XPS spectra of Ba 3d^5/2^ (**a**), Mn 2p^3/2^ (**b**), and Mn 3p (**c**). XPS spectra of O 1s (**d**), Cu 2p^3/2^ (**e**), and Cu L_3_M_4.5_M_4.5_ (**f**) transitions.

The XPS profiles corresponding to Ba 3d^5/2^, Mn 2p^3/2^, Mn 3p, Cu 2p^3/2^, and O 1s transitions are presented in Figure 2a–f, and the most relevant XPS data are included in Table 4 and Table 5. In Figure 2, the XPS profiles of BM perovskite [32] were included for comparison.

The analysis of the Ba 3d^5/2^ transition (Figure 2a) for all samples reveals a major contribution from lattice barium compared to Ba species linked to carbonate groups. On the other hand, by deconvolution of the Mn 2p^3/2^ transition (Figure 2b), two components are found: (i) one located at the lowest binding energy (between 641 and 641.5 eV), which is associated with Mn(III), and (ii) one detected around 643 eV, related to Mn(IV). An additional contribution has been detected between 644 and 645 eV, which corresponds to the satellite peak due to the presence of Mn(III) on the catalysts surface [54,55]. Comparing the spectra of copper-doped samples with the corresponding BM perovskite, it is observed that the presence of the dopant causes a chemical shift of the Mn signal towards higher binding energies. Thus, considering that Cu presents a higher electronegativity that Mn, this fact should be due to an electronic transfer from Mn to Cu that decreases the electron density of Mn. The Mn(IV)/Mn(III) ratio (Table 4), calculated based on the area of the deconvoluted bands for each sample, suggests that in the presence of Cu, the oxidation of Mn(III) to Mn(IV) occurs on the surface, as indicated by the higher values in the presence of Cu dopant for BxMC samples. However, as BMC presents a similar Mn(IV)/Mn(III) ratio to that of BM, this effect seems to be related with the Ba deficiency. This is consistent with the previously observed chemical shift due to the electron transfer from Mn to Cu. A further analysis of these data reveal that the Mn(IV)/Mn(III) ratio increases as the Ba content decreases, so it seems that the oxidation of Mn(III) to Mn(IV) takes place as a charge compensation mechanism on the surface to counteract the deficiency of positive charge. Similar trends have been observed in previous studies, where the positive charge defect generated by the modification of the A cation stoichiometry led to the oxidation of Mn(III) to Mn(IV) on the surface [56,57,58].

However, it has to be underlined that, in the Mn 2p^3/2^ spectra, the binding energies of Mn(III) and Mn(IV) deconvolutions are not different enough to allow for the accurate determination of the oxidation states of Mn, which, in fact, is a complex task [59]. Thus, in order to try to improve the accuracy of the assignment, the analysis of the splitting found in the Mn 3s transition has been proposed [59,60,61], but, for the BxMC sample series, this analysis is hindered by the overlap of Mn 3s with Ba 4d transition [60]. As an alternative, the analysis of the Mn 3p transition has been also suggested [62]; a Mn 3p signal close to 49 eV is detected if Mn(III) is present as the main oxidation state on the surface. Thus, the data in Figure 2c seem to confirm that the incorporation of Cu into the BaMnO_3_ lattice (BMC) did not significantly increase the amount of Mn(IV), and that the Ba deficiency caused an increment of Mn(IV) proportion, since the Mn 3p signal is shifted towards higher binding energy values for BxMC samples.

The deconvolution of the O 1s signal, shown in Figure 2d, reveals the presence of the different contributions usually found in perovskites: (i) a band located at binding energies around 529 eV, corresponding to the lattice oxygen (O_L_) [63]; (ii) a band at around 531 eV, due to the presence of oxygen with low coordination, which corresponds to the oxygen vacancies formed on the surface (O_def_) [64]; and a signal at ca. 532 eV, which features the presence of adsorbed oxygen and hydroxyl and carbonate groups on the catalysts surface (O_ads_) [65,66,67]. According to the XPS profiles, the copper-containing samples exhibit more surface oxygen vacancies than the BM reference sample, indicating that this charge compensation mechanism also takes place as a result of the partial substitution of Mn by Cu and of the Ba deficiency. In Table 4, the XPS and nominal values of the O_L_/(Ba + Mn( + Cu)) ratio are shown. The XPS values have been calculated using the area under the deconvoluted signals, while the nominal values (shown between parentheses in Table 4), are calculated using the molecular formula of samples. Note that an experimental ratio lower than the nominal one indicates the presence of oxygen vacancies (defects) on the surface. Thus, all samples present oxygen surface vacancies, with an increase as the Ba content decreases.

Figure 2e displays the profiles corresponding to the Cu 2p^3/2^ transition. Some of the perovskites exhibit a band at a binding energy lower than 933 eV (except for B0.8MC, whose Cu 2p^3/2^ signal appears at approximately 933.8 eV), which, considering the proposal of Yu et al. [68], indicates the presence of Cu(II) on surface. The location of the XPS signal at binding energy values lower than 933 eV suggests that: (i) the Cu(II) species are located in an electron-rich environment due to a strong interaction with Mn, or (ii) the oxidation state of Cu species is lower than Cu(II). Thus, it should correspond to the presence of Cu(I) or Cu(0). The presence of Cu(II) is supported by the satellite peaks found between 939 eV and 942 eV [69]. Focusing attention on the deconvolution of the Cu 2p^3/2^ band, two contributions have been detected: (i) the signal located at lower binding energies, which corresponds to surface Cu(II) species that interact more strongly with the perovskite (Cu_si_), thus resulting in a higher electronic density; and (ii) the signal at higher binding energies, associated with surface Cu(II), that interacts weakly with the perovskite (labeled as Cu_wi_) [31,70,71]. The proportion of these two contributions, displayed in Table 5 as Cu_si_/Cu_wi_ ratio, states that the fraction of copper species with a strong interaction with the perovskite lattice decreases as the amount of Ba vacancies increases. Thus, the increase in the proportion of Cu_wi_ suggests that Cu is being accommodated in larger sites within the perovskite network, which was one of the hypotheses proposed during the discussion of the XRD results. Additionally, note that the highest Cu_si_/Cu_wi_ ratio found for the BMC sample seems to justify the stabilization of the Cu_16_O_14.15_ phase. The distribution of Cu in the samples can be analyzed by comparing the theoretical (from the perovskite formula) and the experimental (based on the area of XPS deconvoluted peaks) values of the Cu/(Ba + Mn + Cu) ratio (Table 5). If the experimental Cu/(Ba + Mn + Cu) ratio is higher than the theoretical value, it indicates that Cu is accumulating on the surface, and the opposite would indicate that Cu enters the perovskite bulk, either in the lattice or forming larger CuO particles. According to the values in Table 5, the percentages of surface Cu decrease with Ba content, so the fraction of Cu(II) located into the perovskite bulk increases as the Ba content decreases. Thus, the lattice vacancies generated by the Ba deficiency would allow the formation of internal domains of CuO, being in agreement with the higher crystallinity of this species revealed by XRD data.

Finally, a deeper analysis of the Cu oxidation states in BxMC samples was performed using a more sensitive procedure based on the Cu L_3_M_4.5_M_4.5_ Auger peak, which is presented in Figure 2f, including the corresponding kinetic energy values in Table 5. Thus, since the Auger peak for BMC is centered at 918.1 eV, the presence of Cu(II) species [72] is confirmed. For samples with lower Ba contents, the Cu L_3_M_4.5_M_4.5_ peak is slightly shifted towards lower kinetic energies, suggesting the existence of Cu(I) species. This is supported by the decrease in the intensity of Cu(II) satellite peaks, indicating a lower amount of Cu(II) (see Cu_sat_/Cu_2p_ ratio values in Table 5, where Cu_sat_ is the area of the Cu(II) satellites, and Cu_2p_ is the area of the entire Cu 2p^3/2^ band located at approximately 933 eV). For B0.8MC the lowest kinetic energy value (917.5 eV) was found, indicating, according to the literature [72], the presence of Cu_3_O_2_ species (which is a mixed copper oxide containing both Cu(I) and Cu(II) ions, Cu_2_^+^Cu^2+^O_2_).

### 3.3. Reducibility and Redox Properties

**Figure 3:**
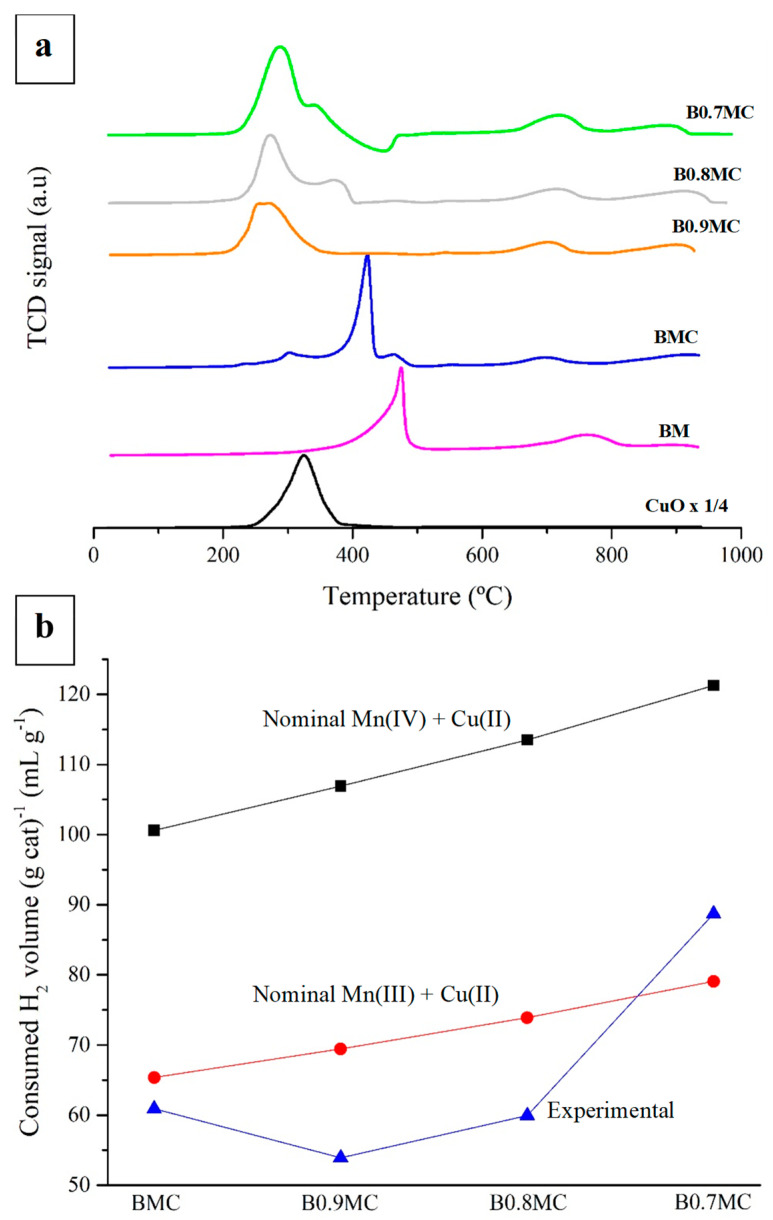
H_2_-TPR consumption profiles of BMC, BxMC samples, and CuO and BM as references (**a**), and H_2_ consumption data (mL H_2_ (g of cat)^−1^) (**b**).

Figure 3a shows the H_2_ consumption profiles obtained for the samples using the H_2_-TPR technique. For manganese-based perovskites, three peaks are usually identified [73,74,75,76]: (i) a low temperature peak, located between 400 °C and 500 °C, assigned to Mn(IV)/Mn(III) reduction to Mn(II); (ii) a low intensity signal between 700 °C and 800 °C, corresponding to the reduction of oxygen species from perovskites; and (iii) another very low intensity peak at approximately 900 °C, due to the reduction of bulk Mn(III) to Mn(II). Furthermore, for these samples, the reduction of Cu(II) to Cu(0), which occurs at temperatures between 230 °C and 400 °C, must be considered, in line with the CuO profile included in Figure 3a. Additionally, to determine the effect of the Cu doping on the reducibility of samples, the profile for BM perovskite was included.

First of all, the BMC profile is similar to that of the BM sample, although the most intense peak is shifted towards lower temperatures. Additionally, at temperatures around 300 °C and 470 °C, two very low-intensity H_2_ consumption peaks become visible. Comparing the temperature of the low-intensity peak at 300 °C with that of the CuO reference, it seems that this peak corresponds to the reduction of the CuO phase previously detected by XRD. On the other hand, the low-intensity peak at around 470 °C likely corresponds to a fraction of slightly less reducible Mn species associated to the reduction of Mn(IV) and Mn(III) in the BM perovskite. As already mentioned, the highest intensity reduction peak of the BxMC samples is located at lower temperatures than those corresponding to BM. Several studies reveal that the addition of Cu to certain perovskite formulations improves the reducibility of perovskites due to a Mn-Cu synergistic effect [77,78]. In the case of BxMC samples, the Mn-Cu synergistic effect favors the reduction of Mn, which occurs at lower temperatures than in BM reference sample. Tarjomannejad et al. [78] attribute the improvement in reducibility to the weakening of the Mn-O bond due to the partial substitution of Mn by Cu, which increases the generation of oxygen vacancies in the bulk, allowing a greater ionic mobility that facilitates the reduction processes. Furthermore, in the Ba-deficient perovskites, the most intense reduction peaks appear at lower temperatures, which can be related to the generation of oxygen vacancies. Finally, it is detected that, as the Ba content decreases, the reduction peaks corresponding to CuO and Mn species become progressively better differentiated. In fact, as the reduction peak corresponding to the tenorite phase appears in the same temperature range as the CuO pattern, it likely corresponds to the reduction of CuO species. Thus, the reduction of Mn is disfavored for BxMC samples, probably due to a decrease in the Mn-Cu synergistic effect as a consequence of the lower amount of Ba, which seems to lead to a weaker interaction between Mn and Cu. Thus, as reported by other authors [79,80], the formation of Ba vacancies, which allowed the generation of internal CuO domains (as observed by the XRD and the XPS results), originate a less intimate contact between the Mn and Cu species that decreases the intensity of the observed synergistic effect.

In Figure 3b, the experimental and theoretical volumes of H_2_ consumed per gram of sample are compared. For quantification, Cu(II) oxidation state has been assumed, and its contribution to H_2_ consumption has been taken into account. It is observed that, for all samples, Mn(III) seems to be the main oxidation state in the bulk, as the experimental data are closer to the Mn(III) + Cu(II) theoretical line, although the amount of Mn(IV) seems to be higher for B0.7MC sample. Thus, these results suggest that the decrease in Ba content favors the oxidation of Mn(III) to Mn(IV) in the bulk of perovskite to compensate for the positive charge deficit.

**Figure 4:**
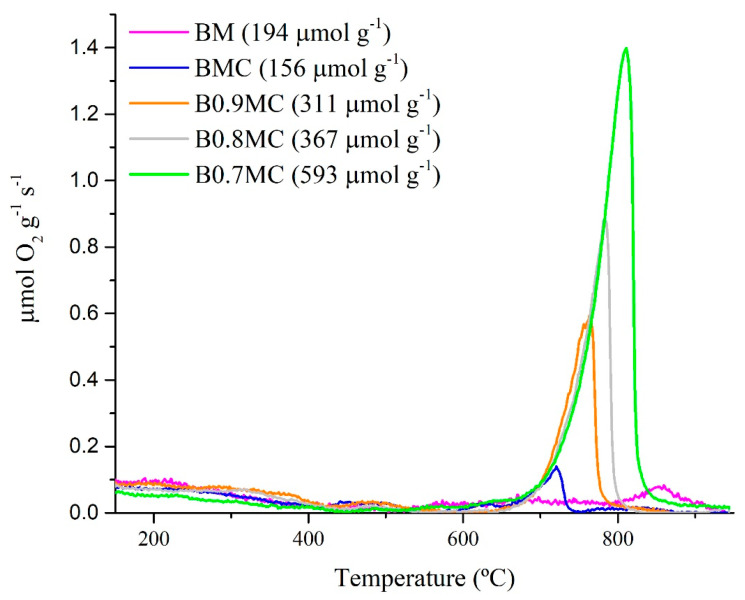
O_2_-TPD profiles of BM, BMC, and BxMC samples.

The oxygen emission profiles as a function of temperature of BMC and BxMC samples are shown in Figure 4, where the BM perovskite has been also included as reference. For perovskites, three contributions are usually observed [81,82,83]: (i) a low-temperature peak between 150 °C and 350 °C, due to the desorption of oxygen adsorbed on surface vacancies (α-O_2_); (ii) an intermediate-temperature signal from 350 °C to 700 °C, corresponding to the desorption of oxygen from the defects that are located in the lattice (α′-O_2_); and (iii) a high-temperature peak, above 700 °C, assigned to the release of lattice oxygen (β-O_2_). According to the Kröeger–Vink reaction (Reaction (9)) [84], the emission of β-O_2_ depends on the reduction of Mn(IV) to Mn(III) and on the presence of bulk oxygen vacancies:

(9) $$2Mn_{Mn}^{x}+O_{O}^{x}⇆2Mn\prime_{Mn}+V_{O}^{••}+1/2\;O_{2}$$

Thus, the temperature at which the peak is detected informs the lattice oxygen mobility through the perovskite network, which is related to the oxidation ability [81]. In Reaction (5), Mn^x^_Mn_ and Mn′_Mn_ correspond to lattice Mn(IV) and Mn(III), respectively, O^x^_O_ represents the oxygen atoms located in an oxygen site, and V^••^_O_ indicates the oxygen vacancies with a double electron deficiency. Additionally, as previously stated for the Cu-dopped samples, it is necessary to consider the contribution of Cu(II) to Cu(I) reduction, according to Reaction (10):

(10) $$2{Cu}_{Cu}^{x}+O_{O}^{x}⇆2Cu\prime_{Cu}+V_{O}^{••}+1/2\;O_{2}$$

Cu^x^_Cu_ and Cu′_Cu_ represent the Cu(II) and Cu(I) species in the perovskite lattice. Note that all samples exhibit a significant lattice oxygen emission which increases as the Ba content decreases and that a lower amount is evolved by stoichiometric BMC and BM samples, being slightly lower for BMC. This trend seems to indicate that the amount of Mn(IV) and of the oxygen vacancies in the bulk of perovskites increases as Ba content decreases; meanwhile, they are lower for BM and BMC. It is remarkable that this sequence completely agrees with the results detected for the surface, as revealed by XPS data (see Table 4). Additionally, a shift in the temperature for the maximum of oxygen emission is evident, so the process is facilitated for the Cu-doped perovskites BMC and BxMC, revealing a synergy between Mn(IV)/Mn(III) and Cu(II)/Cu(I) redox pairs. Finally, the generation of oxygen seems to also improve by the Ba deficiency due to the larger amount of vacancies generated in the bulk.

### 3.4. Electrical Properties

**Figure 5:**
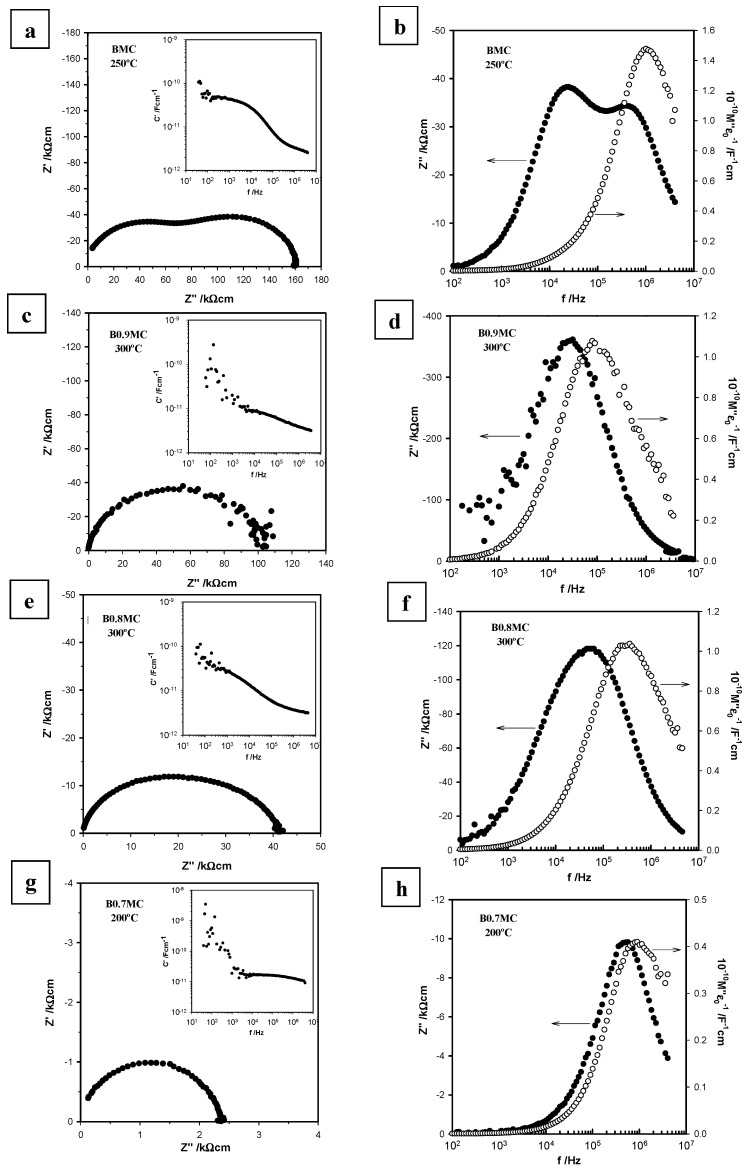
Impedance complex plane plots (**a**,**c**,**e**,**g**), C′ spectroscopic plots (insets) and Z″/M″ spectroscopic plots (**b**,**d**,**f**,**h**) at temperatures between 200 and 300 °C for BMC and BxMC samples.

Typical results of impedance measurements at several temperatures on pellets sintered at 800 °C for 2 h in dry N_2_ are shown in Figure 5 for the BMC and BxMC samples. In the case of the samples showing the BaMnO_3_ polytype as the main phase (BMC, B0.9MC, and B0.8MC), impedance complex plane plots, Z* (Figure 5a,c,e), show the presence of a broad and depressed semi-circular arc, which is more evident for the stochiometric sample (BMC), suggesting the existence of multiple RC (resistive–capacitive) elements associated in series. The log C′ vs. log f plots, inserted in these figures, reveal the presence of two dispersions at high and low frequencies. The high-frequency plateau with capacitance values ~2.8–3.4·10^−12^ F cm^−1^, is attributed to the bulk capacitance, whereas the poorly resolved low frequency plateau at ~1–4·10^−11^ F cm^−1^, is smaller than expected for a grain boundary or surface layer [42] but represents an additional element with a small volume fraction resistance that contribute to the total resistance.

To clarify the possible presence of two different RC elements in these samples, the Z″/M″ spectroscopic plots are shown in Figure 5b,d,f. The B_x_MC samples show that the Z″ and M″ peak maxima do not match at the same frequency maximum. Additionally, the Z″ peak exhibits a broader format than expected for a Debye peak. This behavior suggests a potential electrical inhomogeneity within the sample with more than one region contributing to the total conductivity, which becomes more prominent for the stoichiometric sample, BMC. This effect can be induced by a combination of either a constriction grain boundary or possible inhomogeneous distribution of the ions in the sample, as well as the presence of the other minority secondary phases.

Regarding the sample with the highest Ba deficiency, B0.7MC, for which BaMnO_3_ hexagonal is the main phase, the impedance complex plane plot (Figure 5g), exhibits a single and almost symmetric semi-circular arc, which can be associated to a single resistance of the sample bulk. The log C′/log f plot inserted in this Figure 5g reveal the presence of an almost frequency-independent plateau associated with the bulk capacitance of the sample, with a capacitance value of 0.12·10^−12^ F cm^−1^. For this sample, the maximum peak frequencies of Z″ and M″ are almost coincident. It is important to note that although a single Debye peak is observed in Z″ and another plateau at low frequencies in the C′ plot is not observed, a broader M″ peak is denoted, in particular at high frequencies. It could be related to the presence of a minority phase in the B0.7MC sample.

Finally, the impedance results do not show any resistance associated with ionic transport or a Warburg impedance, typically represented by a spike at low frequencies. This observation, combined with the low capacitance values (10^−9^–10^−8^ F cm^−1^) at low frequencies compared to the typical values for ionic conductors (which are around 10^−6^ F cm^−1^), confirms that the electronic conduction mechanism dominated the response in the BMC and BxMC samples.

**Figure 6:**
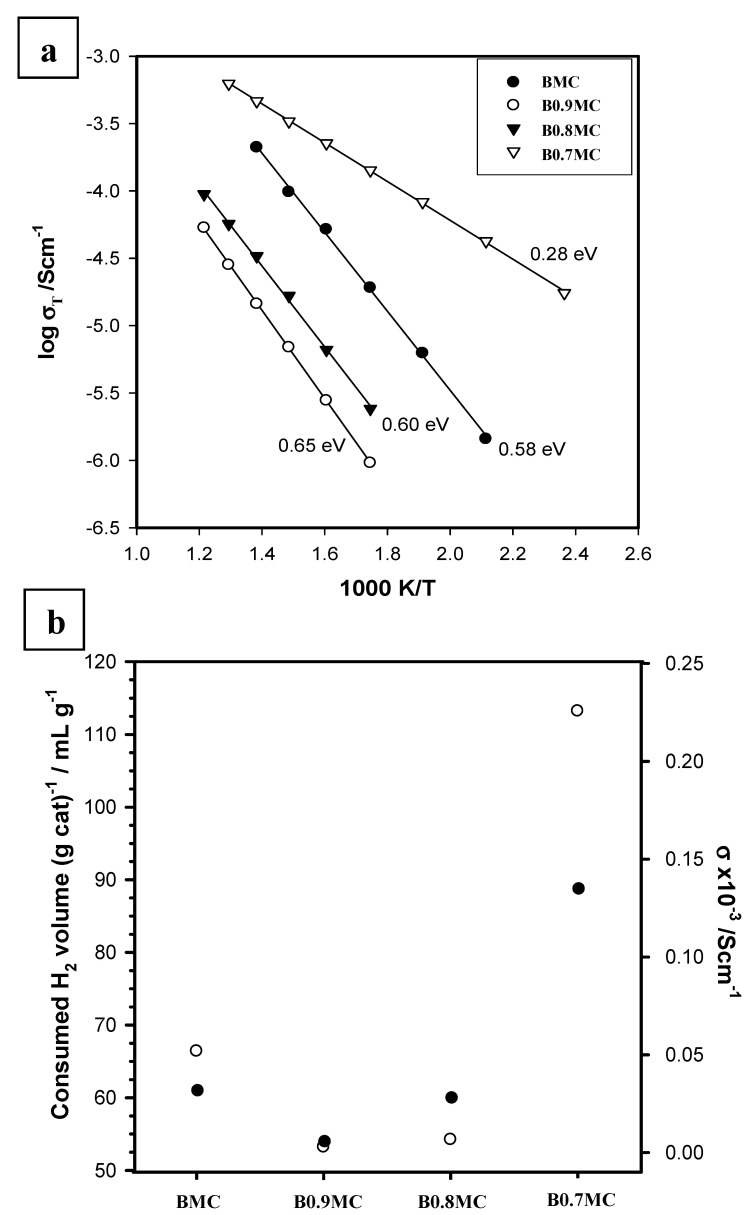
(**a**) Arrhenius plots of total conductivity for BMC and BxMC samples in a dry N_2_ atmosphere. The activation energy (eV), with errors in the range 0.02–0.05 eV, are included for each dataset. (**b**) Total conductivity at 350 °C in dry N_2_ (black points) and H_2_ volume consumed (white points) as a function of the composition.

The total resistance of each composition was obtained from intercepts of the arc on the Z′ axis of the impedance complex plan plots, and the conductivity was plotted in Arrhenius format as a function of temperature, as seen in Figure 6a. From this plot, the corresponding activation energies were calculated, employing Equation (11):

(11) $$\sigma =\sigma_{0}e^{\left(-\frac{E_{a}}{kT}\right)}$$

The impedance measurements at different temperatures were performed after the system reached steady state conditions. The activation energies are also shown beside each dataset.

The total conductivity decreases for the B0.9MC sample respect to BMC and, subsequently, an increase with the Ba deficiency is observed, with the B0.7MC sample being the most conductive one. As the number of Ba defects increases, a decrease in the activation energy is found, suggesting a change in the conduction mechanism, which can be associated with the presence of different crystalline phases.

Due to the deficiency in A-site cations of perovskite, a lower resistance for BxMC samples would be expected, but it is observed that the total resistance increases for x = 0.9 and 0.8 compositions. It is important to note that impedance measurements are highly sensitive to the presence of grain boundaries and to the heterogeneity of the crystalline structure [85]. Thus, keeping in mind the XRD results previously discussed, it seems that:

(i) BMC and B0.7MC samples show higher conductivities as an almost unique crystalline phase (BaMnO_3_ polytype for BMC and hexagonal BaMnO_3_ for B0.7MC) is detected, presenting BMC having a higher resistance than B0.7MC, probably due to the contribution of the other minority phases and/or to the disorder degree of the polytype structure.
(ii) For B0.9MC and B0.8MC samples, the polytype and the hexagonal perovskite structures coexist, causing the observed increase of the resistivity respect to BMC and B0.7MC.

Finally, as it could be expected, the reducibility of the samples could be related to their conductivity, and therefore a correlation between the conductivity and the reducibility of samples is observed in Figure 6b, where the experimental (consumed) H_2_ volume is plotted against the total conductivity at 350 °C for all samples. The B07MC sample with a hexagonal perovskite structure has a higher conductivity than the other samples that present the polytype structure. This trend is also observed in the value of the volume of hydrogen consumed.

In summary, it appears to be confirmed that the conductivity of the samples, which depends on their crystalline structure, is linked to their reducibility, and is therefore expected to play a significant role in their catalytic performance.

### 3.5. Catalytic Activity

**Figure 7:**
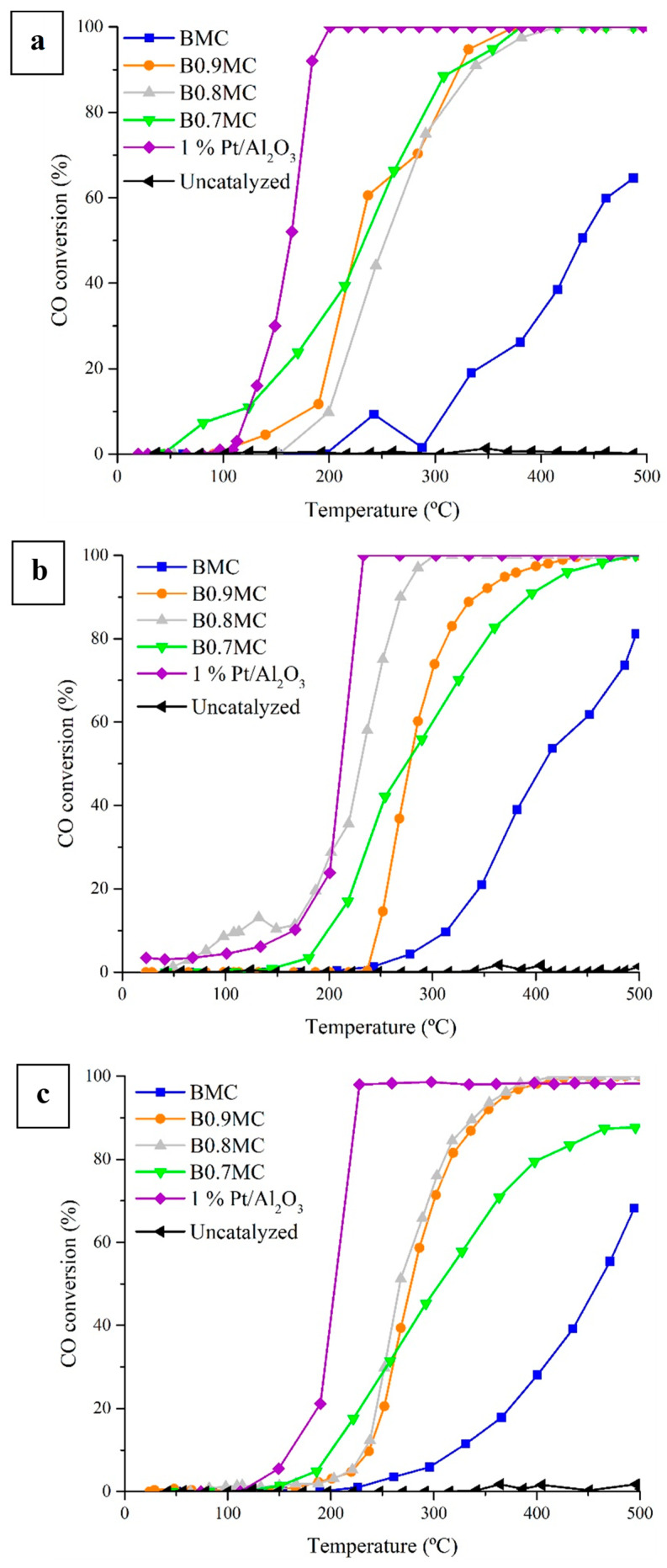
CO conversion profiles of BMC, BxMC, and 1% Pt/Al_2_O_3_ samples under 0.1% CO/1% O_2_/He (**a**); 1% CO/1% O_2_/He (**b**); and 1% CO/10% O_2_/He (**c**) reactant mixtures.

Catalytic CO oxidation experiments in Temperature-Programmed Reaction conditions (CO-TPRe) were carried out to evaluate the catalytic performance of BMC and BxMC samples. Three reactant mixtures with CO:O_2_ ratios of 0.1:1, 1:1, and 1:10 were used, with the corresponding CO conversion profiles shown in Figure 7 and the T_50%_ values (T_50%_ is the temperature to achieve a 50% of CO conversion) featured in Table 6. Both the conversion profiles and T_50%_ were compared with those obtained for a 1% Pt/Al_2_O_3_ catalyst, used as reference.

First of all, all samples (BMC and BxMC) are able to catalyze the CO oxidation reaction in the tested conditions and BMC is the less active sample. If the most favorable composition (0.1% CO, 1% O_2_) is used, the three Ba-deficient samples show a similar improvement of the CO conversion, featuring T_50%_ values lower than for BMC. However, for the other two atmosphere compositions (CO:O_2_ of 1:1 or 1:10), the Ba deficient samples present different performances, that is, different CO conversion profiles and T_50%_ values. In general terms, the formation of oxygen vacancies on the surface (see the O_L_/(Ba + Mn + Cu) ratios in Table 4) and the location of the copper species in the inner sites of the perovskite network (according to the decrease of the Cu/(Ba + Mn + Cu) values shown in Table 5) allows improvement of the catalytic performance of the samples, even though the Mn-Cu synergistic effect is less intense for the BxMC samples. Specifically, B0.8MC presents the lowest T_50%_ value (see Table 6), close to that found for the 1% Pt/Al_2_O_3_ reference sample for the 1:1 atmosphere. According to the characterization results, the B0.8MC sample features a high Mn(IV)/Mn(II) surface ratio, the highest amount of oxygen vacancies, and the highest fraction of reduced copper species that, according to the literature [86,87], are more active for CO oxidation than CuO in the presence of oxygen and at a temperature higher than 200 °C. Thus, independently of the less intense Mn-Cu synergistic effect, it is evident that the specific interaction between Mn and Cu species in the B0.8MC sample ensures the presence of a higher amount of Cu(I) species, originated together with the oxidation of Mn(III) to Mn(IV) (as seen in XPS characterization), which promotes the formation of oxygen defects (supported by the data featured in Table 4 and also reported by other authors [88]). Thus, in the 1:1 atmosphere, it is expected that CO and O_2_ molecules would be adsorbed on Cu(I) sites, with this oxidation state reduced to Cu(0), which is also active for CO oxidation at high temperatures through a Langmuir–Hinshelwood mechanism [86,87]. On the other hand, during the reaction under 1:10 atmosphere, Cu(I) species would be oxidized to Cu(II), which is not able to be reduced to Cu(0) at high O_2_ partial pressures [86]. Thus, in the 1:10 atmosphere, the T_50%_ value is higher than in the 1:1 atmosphere. Finally, it is necessary to take into account that Cu(I) is able to dissociate the O_2_ molecule, but Cu(II) is not (due to its insulating properties [87]).

**Figure 8:**
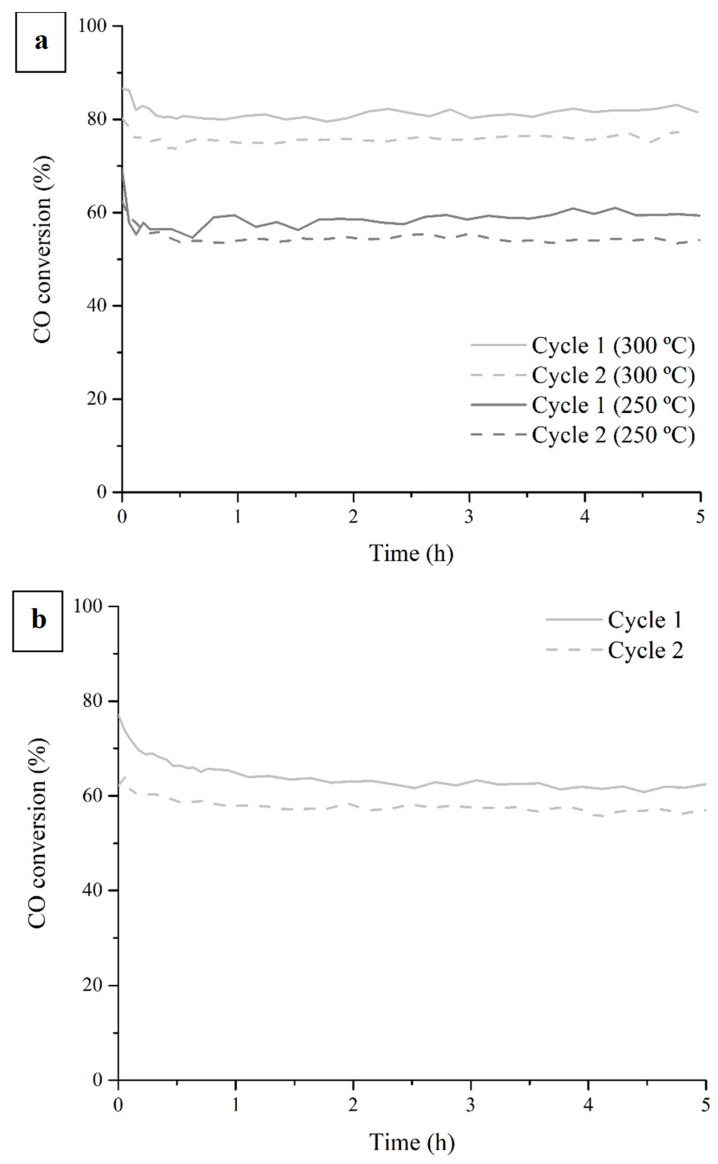
CO oxidation profiles of B0.8MC sample at 250 °C and 300 °C under 1:1 reactant mixture (**a**) and at 300 °C under 1:10 atmosphere (**b**).

As the B0.8MC sample is the most active sample for CO oxidation reaction in the most demanding conditions (1:1 and 1:10 atmospheres), it has been selected to carry out stability tests consisting of two consecutive reaction cycles developed with the following conditions: (i) at 300 °C, over 5 h, in 1:1 and 1:10 reactant mixtures, and (ii) at 250 °C, over 5 h, in the 1:1 reactant mixture. The CO conversion profiles are featured in Figure 8, and the corresponding conversion related data (the deactivation degree and CO specific activity) are displayed in Table 7. According to these results, the B0.8MC sample showed a stable performance during 5 h at 300 °C in the 1:1 atmosphere, while the CO conversion decreased if 1:10 reactant mixture was used, with deactivation values of 19% and 8% for this composition, versus 8% and 1% for the 1:1 reactant mixture. Note that, because of the lower CO conversions for the 1:10 atmosphere, the CO specific activity values are lower than the observed in the 1:1 reactant mixture. Finally, at 250 °C, similar to the observation at 300 °C, a great stability during 5 h of reaction time is shown.

**Figure 9:**
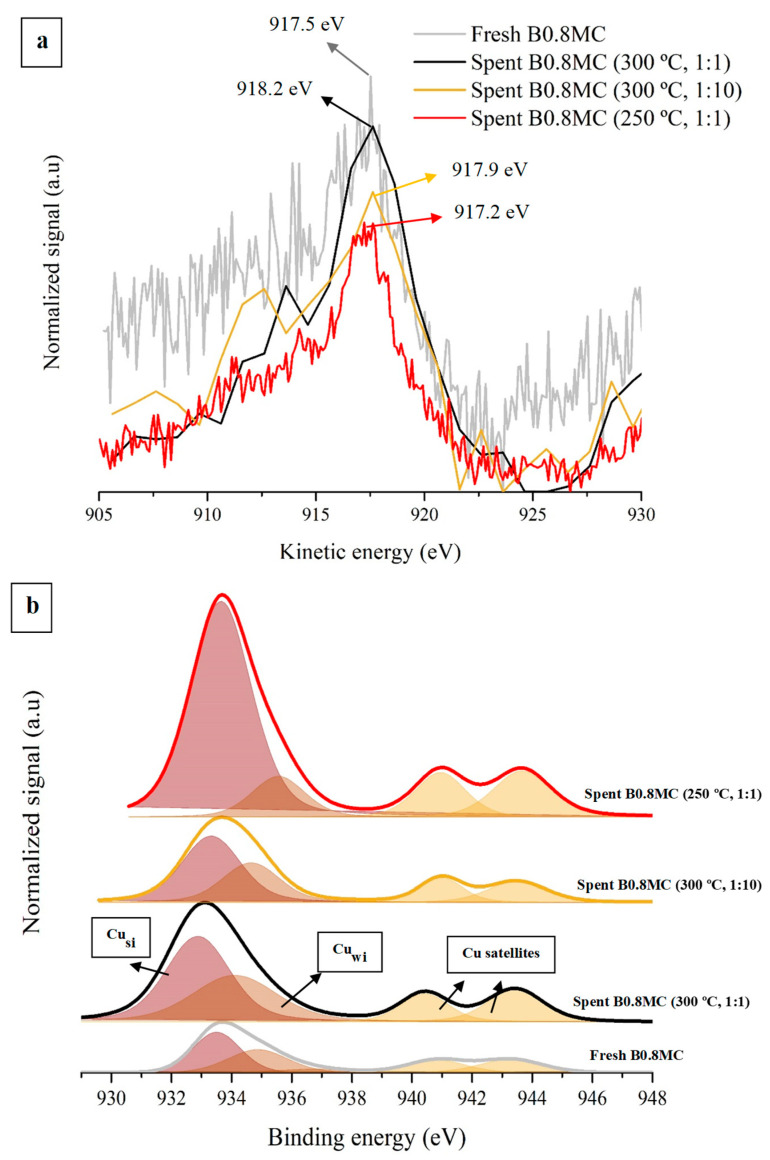
Cu L_3_M_4.5_M_4.5_ Auger peaks (**a**) and Cu 2p^3/2^ spectra (**b**) of the fresh and spent (at 250 °C and 300 °C) B0.8MC samples.

On the other hand, note that for the two atmospheres (1:1 and 1:10) and reaction temperatures tested (250 and 300 °C), the CO conversion values are lower during the second cycle than during the first one, with a lower decrease at 250 °C. In order to justify the decrease in CO conversion during the second cycle, the samples used in the stability tests were characterized by XRD and XPS. Table 8 presents the XPS data obtained for the used samples, including the data of the fresh sample for an easier comparison. The BaCO_3_/Ba_L_ ratio indicates that all used samples present a similar or higher carbonation degree compared to the fresh one, which affects the stability of the B0.8MC sample. This is because, as reported in the literature, carbonate groups cover the available active sites (where the reactant molecules are activated) and, consequently, cause the deactivation of the catalysts [89,90,91]. Additionally, for used samples, the Mn(IV)/Mn(III) ratio decreases in all tested conditions and, consequently, the amount of oxygen defects increased. The generation of more defects on the surface at 300 °C may be caused by the migration of Cu ions from the surface to the bulk, since the Cu/(Ba + Mn + Cu) ratio decreases. However, at 250 °C, an increase of the Cu/(Ba + Mn + Cu) ratio is detected, suggesting an exsolution of copper from the inner layers of solid to the surface [92]. Regarding the evolution of the Mn(IV)/Mn(III) ratio, the decrease of the amount Mn(IV) species is linked to the different distribution of Cu after the stability tests, since a decrease of the Mn-Cu interaction is produced. On the other hand, the Cu L_3_M_4.5_M_4.5_ Auger peaks of the used samples were analyzed to determine the possible changes in the oxidation state of the copper species produced by the loss of interaction with the Mn species, and by the interaction with the reactants, with the results presented in Figure 9a. As previously described, the fresh B0.8MC sample presents a fraction of reduced copper species on surface (see Figure 2f). For the B0.8MC sample used at 300 °C, a shift towards higher kinetic energies is observed in both Cu L_3_M_4.5_M_4.5_ Auger signals. In the 1:1 atmosphere, the Auger signal is located at a kinetic energy value of 918.2 eV, corresponding to Cu(0) [72,93], while in the 1:10 reactant mixture, the Auger peak location (917.9 eV) suggests the presence of Cu(II) [72,93]. Thus, Cu(0) and Cu(II) coexist on the surface of the former sample (considering the presence of the Cu(II) satellites in the Cu 2p^3/2^ transition, shown in Figure 9b), but not for the latter sample. This finding agrees with the expected results for a more oxidizing atmosphere in which the Cu(II) should be favored [86], and it seems to explain the decrease in the catalytic activity observed in the 1:10 conditions. Thus, the excellent stability of B0.8MC under 1:1 atmosphere can be attributed to the high fraction of reduced copper species present on the surface during and after the reaction. Regarding the registered Auger signal for the sample used at 250 °C, its position (917.2 eV) reveals the presence of Cu(I) species [72] which, of course, coexists with Cu(II). Thus, the great stability of B0.8MC in these conditions seems to be justified.

**Figure 10:**
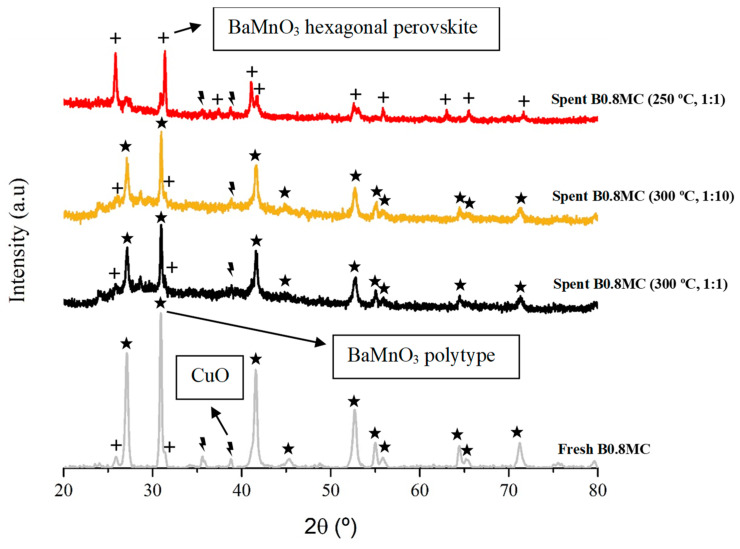
XRD patterns of fresh and used (at 250 °C and 300 °C) B0.8MC samples.

Finally, the XRD profiles of the fresh and used B0.8MC samples are displayed in Figure 10, where a mixture of the three crystalline phases (BaMnO_3_ perovskite, BaMnO_3_ polytype, and CuO) are identified. Regarding the sample used at 300 °C, it is observed that the BaMnO_3_ polytype structure is still the major one and the CuO peaks are less clearly identified due to their lower intensity. This observation agrees with the decrease in the Cu/(Ba + Mn + Cu) ratio that suggests the partial introduction of the Cu species into the inner layers of the solid. In the case of the sample used at 250 °C, a transition of the polytype to the hexagonal perovskite structure is observed, confirming that Cu species were exsoluted to the surface and, consequently, the distortion of the perovskite structure is less significant.

## 4. Conclusions

In this work, a series of Ba_x_Mn_0.7_Cu_0.3_O_3_ (BxMC, x = 1, 0.9, 0.8, and 0.7) perovskites were synthesized, characterized, and tested for CO oxidation reaction under simulated gasoline exhaust conditions. The main conclusions are as follows:

- The Ba content in BxMC perovskites formulations determines the Cu distribution and, consequently, the structure of the samples.
- The amount of Mn(IV) and of oxygen vacancies (defects) increases as the Ba content decreases, and B0.8MC exhibits an enrichment of reduced copper species on the surface.
- In the presence of Cu, a Mn-Cu synergistic effect is observed, which promotes the reducibility and the oxygen emission. However, this synergistic effect decreases with the Ba content, due to the different location of Cu into the perovskite network.
- The reducibility trend during the H_2_-TPR characterization tests appears to be directly linked to the total conductivity of perovskites.
- All BxMC perovskites tested are active for CO oxidation, showing B0.8MC the best catalytic performance, which is the closest to that of 1% Pt/Al_2_O_3_ reference sample. The outstanding catalytic performance of B0.8MC is mainly related to the presence of a high Mn(IV)/Mn(III) ratio and the highest amount of oxygen vacancies and of reduced copper species on surface.
- A correlation is observed between the conductivity, the crystalline structure, and the reducibility of the samples, which is, in turn, related to their catalytic performance.
- The stability of B0.8MC during the isothermal reaction depends on the temperature and on the reactant atmosphere composition (CO and O_2_ proportion). Thus, the reaction temperature determines the distribution of copper species in the structure and the stability is highly dependent on the proportion of reactants, with the preservation of the reduced copper species (Cu(I) and Cu(0)) on the surface in the most reductant atmosphere composition (CO:O_2_ 1:1) being the key factor, as these oxidation states are more effective for CO and O_2_ activation than Cu(II).

As stated in the present study, the catalytic performance of the Ba_x_Mn_0.7_Cu_0.3_O_3_ formulations was evaluated under simulated exhaust conditions based on CO + O_2_ simple atmospheres. In this context, the B0.8MC sample will be selected, as it is one of the best formulations developed by the authors, to be tested under a more complex CO_2_-containing atmospheres, because the tolerance to CO_2_ is a key property that the catalysts must present. Thus, it is expected that a future publication will focus on this topic, in which the optimization of the formulations required to improve the tolerance to CO_2_ will be considered.

## Figures and Tables

**Table 1:** Specific surface area and copper content of BxMC samples.

Sample	Nomenclature	BET Surface Area (m^2^ g^−1^)	Nominal Cu (wt %)
BaMn_0.7_Cu_0.3_O_3_	BMC	1	8
Ba_0.9_Mn_0.7_Cu_0.3_O_3_	B0.9MC	6	8
Ba_0.8_Mn_0.7_Cu_0.3_O_3_	B0.8MC	7	9
Ba_0.7_Mn_0.7_Cu_0.3_O_3_	B0.7MC	4	9

**Table 2:** XRD data of BxMC samples and BM perovskite as reference.

Sample	Cell Parameters (Å) ^1^		Perovskite Average Crystal Size (nm)	(110) Interplanar Spacing (Å)	Lattice Strain
	a	c			
BM	5.7	4.8	24.8	2.85	3.0·10^−4^
BMC	5.8	4.3	57.8	2.88	2.1·10^−3^
B0.9MC	5.8	4.3	29.5	2.89	1.1·10^−3^
B0.8MC	5.8	4.4	81.6	2.89	3.9·10^−3^
B0.7MC	5.7	4.8	53.3	2.85	1.6·10^−3^

^1^ Considering that the relationship between the cell parameters in the hexagonal crystal system is a = b ≠ c, only a and c parameters are included in this table.

**Table 3:** XRD refinement data.

Sample	Hexagonal BaMnO_3_ (%)	Polytype BaMnO_3_ (%)	CuO/Cu_16_O_14.15_ (%)	Ba_3_Mn_2_O_8_/BaMn_8_O_16_ (%)
BM	98	-	-	2
BMC	-	96	3	1
B0.9MC	3	93	4	-
B0.8MC	8	85	7	-
B0.7MC	82	8	10	-

**Table 4:** Mn and O XPS characterization data of fresh BxMC samples (BM was included as reference).

Sample	Mn(IV)/Mn(III)	O_L_/(Ba + Mn + (Cu)) (Nominal)
BM	0.2	1.2 (1.5)
BMC	0.3	0.8 (1.5)
B0.9MC	0.8	0.9 (1.6)
B0.8MC	1.0	0.9 (1.7)
B0.7MC	1.1	1.0 (1.8)

**Table 5:** Cu XPS characterization data of fresh BxMC samples.

Sample	Cu/(Ba + Mn + Cu) (Nominal)	Surface Cu (%) ^1^	Cu_sat_/Cu_2p_	Cu_si_/Cu_wi_	Cu 2p^3/2^ BE (eV)	Cu LMM KE (eV)
BMC	0.14 (0.15)	93	0.6	2.7	932.28	918.12
B0.9MC	0.09 (0.16)	56	0.5	2.0	932.88	917.62
B0.8MC	0.08 (0.17)	47	0.5	1.4	933.68	917.52
B0.7MC	0.07 (0.18)	39	0.4	1.2	933.28	917.72

^1^ Calculated by obtaining the percentage between the XPS Cu/(Ba + Mn + Cu) ratio and the theoretical one.

**Table 6:** T_50%_ data of BxMC and 1% Pt/Al_2_O_3_ samples.

Sample	T_50%_ (°C)		
	0.1% CO/1% O_2_/He	1% CO/1% O_2_/He	1% CO/10% O_2_/He
BMC	438	408	459
B0.9MC	227	278	278
B0.8MC	253	231	267
B0.7MC	233	275	306
1% Pt/Al_2_O_3_	164	212	204

**Table 7:** Deactivation and CO specific activity data of B0.8MC sample.

	Atmosphere 1:1 300 °C		Atmosphere 1:10 300 °C		Atmosphere 1:1 250 °C	
	Cycle 1	Cycle 2	Cycle 1	Cycle 2	Cycle 1	Cycle 2
Deactivation (%) ^1^	8	1	19	8	14	13
CO specific activity (5 h) ^2^	0.4		0.3		0.3	

^1^ Calculated as the percentage obtained from the difference between the initial and final CO conversions, divided by the initial conversion. ^2^ As mol of CO converted per mol of Cu and per minute. The actual percentage of Cu (Table 1) has been considered.

**Table 8:** XPS characterization data of the fresh and used B0.8MC samples.

Sample	Mn(IV)/Mn(III)	Cu/(Ba + Mn + Cu)	O_L_/(Ba + Mn + Cu)	BaCO_3_/Ba_L_ ^1^
Fresh	1.0	0.08	1.12	0.2
Used (300 °C, 1:1)	0.6	0.07	0.14	0.7
Used (300 °C, 1:10)	0.6	0.06	0.17	0.4
Used (250 °C, 1:1)	0.6	0.09	0.20	0.2

^1^ Obtained considering deconvolutions of Ba 3d^5/2^ transition.

## Data Availability

Data will be made available on request.
